# Influence of two anti-fungal *Lactobacillus fermentum-Saccharomyces cerevisiae* co-cultures on cocoa bean fermentation and final bean quality

**DOI:** 10.1371/journal.pone.0239365

**Published:** 2020-10-01

**Authors:** Edwina Romanens, Vasilisa Pedan, Leo Meile, Susanne Miescher Schwenninger

**Affiliations:** 1 Research Group Food Biotechnology, Institute of Food and Beverage Innovation, Zurich University of Applied Sciences (ZHAW), Wädenswil, Switzerland; 2 Laboratory of Food Biotechnology, Institute of Food, Nutrition and Health, ETH Zurich, Zurich, Switzerland; 3 Research Group Food Chemistry, Institute of Food and Beverage Innovation, Zurich University of Applied Sciences (ZHAW), Wädenswil, Switzerland; Universite d'Orleans, FRANCE

## Abstract

The growth of filamentous fungi during the spontaneous cocoa bean fermentation leads to inferior cocoa bean quality and poses a health risk for consumers due to the potential accumulation of mycotoxins. We recently developed anti-fungal cultures with the capacity to inhibit the growth of mycotoxigenic filamentous fungi on cocoa beans. However, it is not clear how these anti-fungal cultures affect the fermentation process and cocoa bean quality. For that, the anti-fungal co-cultures, *Lactobacillus fermentum* M017-*Saccharomyces cerevisiae* H290 (A) and *Lb*. *fermentum* 223-*S*. *cerevisiae* H290 (B), were applied to 180-kg box fermentations in Honduras in three time-independent replications each including a spontaneous control fermentation. The comparison of inoculated and spontaneous fermentation processes revealed that the co-cultures only marginally affected the fermentation process and cocoa bean quality. Microorganisms reached maximal levels of 6.2–7.6 log CFU/g of yeasts and acetic acid bacteria and 7.9–9.5 log CFU/g of lactic acid bacteria during all fermentations and led to maximal metabolite concentrations in bean cotyledons of 4–12 mg/g ethanol, 2–6 mg/g lactic acid and 6–14 mg/g acetic acid. The fermentation and drying processes resulted in 38–90 mg epicatechin equivalents/g in the cotyledons of dried beans. However, the co-cultures led to up to ten times higher mannitol levels in cotyledons of inoculated beans compared to beans during spontaneous fermentation, and caused a slower fermentation process, detectable as up to 8–12 °C lower temperatures in the centre of the fermenting pulp-bean mass and up to 22% lower proportions of well-fermented beans after drying. Co-culture B–with *Lb*. *fermentum* 223 –led to improved cocoa bean quality compared to co-culture A–with *Lb*. *fermentum* M017 –, i.e. cocoa beans with 0.5–1.9 mg/g less acetic acid, 4–17% higher shares of well-fermented beans and, on a scale from 0 to 10, to 0.2–0.6 units lower astringency, up to 1.1 units lower off-flavours, and 0.2–0.9 units higher cocoa notes. Therefore, the anti-fungal co-culture B is recommended for future applications and its capacity to limit fungal growth and mycotoxin production during industrial-scale cocoa bean fermentation should be investigated in further studies.

## Introduction

The fermentation of the beans of *Theobroma cacao* L. is the first step in the post-harvest processing of cocoa. It is a spontaneous process in which microorganisms that drive the fermentation originate from the environment [1]. Due to the fact that approximately 80–90% of cocoa produced globally is cultivated on 5–6 million small farms [2], the quality of the resulting cocoa beans is variable. Factors influencing cocoa quality include seasonal variations, changing weather conditions, and different techniques applied during pod-storage, fermentation, and drying [3].

Despite its spontaneous nature, the fermentation of cocoa beans is typically defined by a succession of yeasts, lactic acid bacteria (LAB), and acetic acid bacteria (AAB), as observed for fermentations in different parts of the world [4–7]. The activities of these microorganisms in the pulp result in the production of acids, ethanol, and heat, which diffuse into the bean leading to death of the seed embryo and structural and biochemical changes in the cotyledons [3]. Free amino acids and peptides from enzymatic degradation of proteins and reducing sugars resulting from degradation of sucrose form the precursors of chocolate flavour that result in the typical chocolate flavour upon drying and roasting [8, 9]. Polyphenolic compounds, which impart bitterness and astringency to unfermented cocoa beans, undergo oxidative reactions that lead to a reduction in the content of soluble polyphenols and result in a reduction of bitter and astringent tastes in cocoa beans [10].

In the later fermentation stage, bacilli and filamentous fungi may appear, with the latter being associated with off-flavour production and a public health risk due to the potential accumulation of mycotoxins [11]. Anti-fungal protective cultures have been applied in a series of food fermentations, such as in fermented cereal-based products, sourdough, cheese, or silage, to act specifically against filamentous fungi [12–15]. Recent *in vitro* and i*n vivo* studies have screened LAB and yeast strains for anti-fungal activity against ochratoxin A and aflatoxin producing filamentous fungi with the aim of suppressing their growth and hence leading to lower mycotoxin accumulation in cocoa and coffee bean fermentations [16–20]. However, as far as the authors are aware, anti-fungal cultures have not been applied to industrial-scale cocoa bean fermentation to assess their influence on cocoa bean quality.

During the present study, two anti-fungal LAB-yeast co-cultures were applied to 180-kg cocoa bean box fermentations in Honduras. The strains had previously been selected for their anti-fungal activity against potentially mycotoxigenic filamentous fungi, their metabolite and heat stress tolerance, and their carbon metabolism that is well-adapted to cocoa bean fermentation [19]. The present study aimed to investigate the impact of the protective cultures on the fermentation and drying processes and final cocoa bean quality. For that, microbial counts, physico-chemical parameters, substrates and metabolites, and the polyphenolic contents of inoculated and spontaneous fermentations were compared and the quality of resulting dried beans was assessed with regard to fermentation degree and sensory profile.

## Material and methods

### Anti-fungal co-cultures

Anti-fungal strains used in the present study were produced at industrial scale at Moguntia Schweiz AG (Gossau ZH, Switzerland) and were provided as lyophilised co-cultures, containing 90–97% (w/w) sucrose. Co-culture A consisted of *Lactobacillus fermentum* M017 and *Saccharomyces cerevisiae* H290 and co-culture B of *Lb*. *fermentum* 223 and *S*. *cerevisiae* H290. The culture strains originated from cocoa bean fermentations in Honduras and Brazil and were selected for their anti-fungal activity against mycotoxin-producing filamentous fungi on the surface of cocoa beans and a stress-tolerant and well-adapted metabolism [19]. The three culture strains are stored as cryo cultures in the culture collection of Switzerland (CCOS, Wädenswil, Switzerland) and the lyophilised co-cultures were transported to Honduras on ice and stored at -20 °C until use.

### Procedure of inoculation, fermentation, drying, and sampling

**Fig 1:**
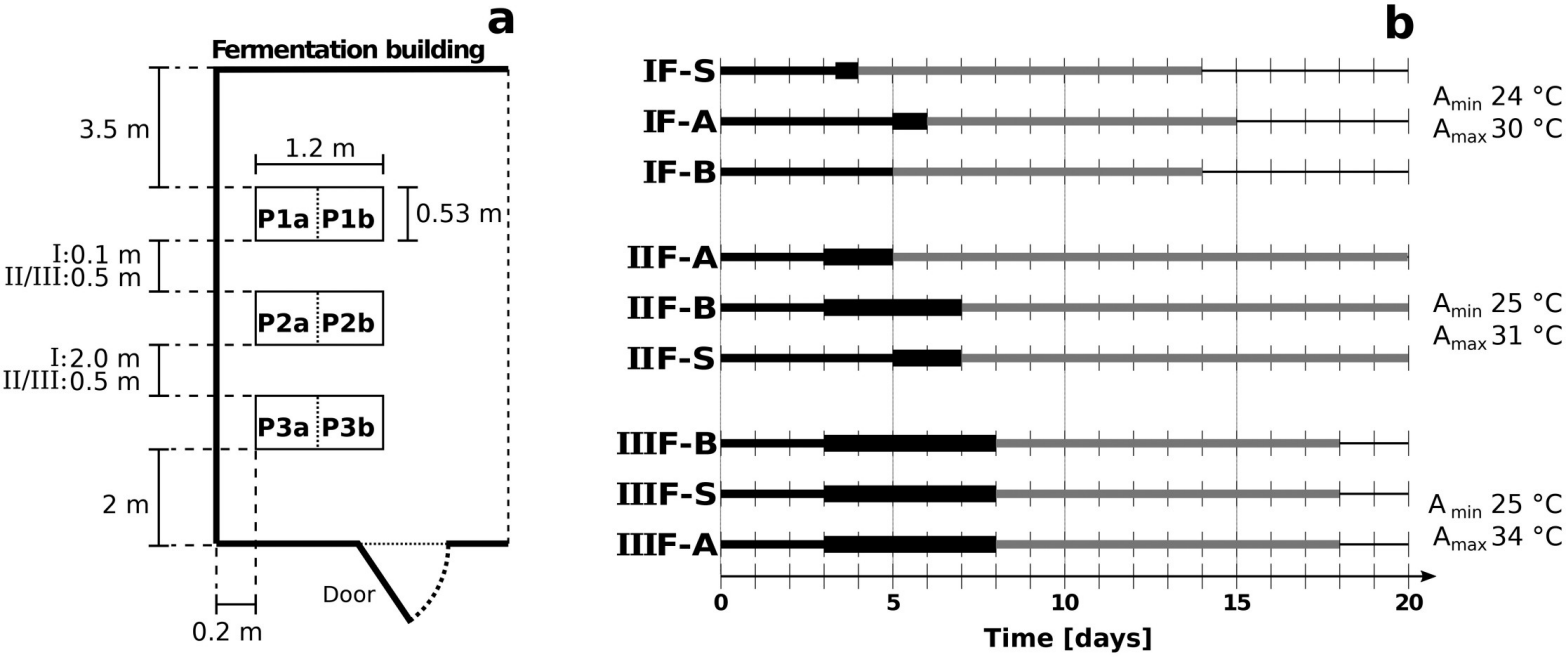
Fermentation building (a), fermentation and drying processes of inoculated and spontaneous fermentations, and ambient temperatures (b). Positions a and b indicate the two parts of the fermentation box in (a). In (b), the positioning of the fermentation boxes F-A, F-B, and F-S during IF, IIF, and IIIF in positions P1-P3 is indicated on the y-axis. The horizontal axis indicates the time of fermentation (black line) and drying (grey line) and time when the fermenting pulp-bean mass in the centre of the box reached temperatures of 45–50 °C (bold black line). P = position; A_min_ = minimal ambient temperature; A_max_ = maximal ambient temperature.

Inoculated fermentations, with co-culture A (F-A) or co-culture B (F-B), and spontaneous control fermentations (F-S) were each carried out as three time-independent replicate fermentations during run I (IF), run II (IIF), and run III (IIIF) in February, March, and April 2017, respectively (Fig 1b). Ripe and healthy cocoa pods of mainly Trinitario hybrids and a smaller quantity of Forastero were harvested in CEDEC (Centro Experimental Demostrativo del Cacao) located in La Masica, Atlántida, Honduras, and opened manually with a machete within 2–3 days for each fermentation run. After separating damaged, mouldy, or overripe beans, pulp-bean mass was collected in bags for weighting. Three wooden boxes measuring 0.6 m x 0.6 m x 0.5 m were each filled with 180 kg of the pulp-beans mass. For IIF and IIIF, the gaps between the wooden planks were closed with wooden slats to minimize heat losses during fermentations. The fresh pulp-bean mass was inoculated within 4 h after its extraction at 6 log CFU/g of *Lb*. *fermentum* M017 or *Lb*. *fermentum* 223 and at 5 log CFU/g of *S*. *cerevisiae* H290. The inoculated pulp-bean mass was covered with banana leaves and the fermentation boxes were transported to FHIA (Fundación Hondureña de Investigación Ágricola) in La Lima, Cortés, Honduras, where the fermentation, the predrying, and the main drying took place.

The position of the fermentation boxes was changed for every fermentation run, so that the boxes of F-A, F-B, and F-S were placed once in each position 1, 2, and 3 in the fermentation building (Fig 1). Mixing and aerating the fermentation mass took place every 24 h by moving the pulp-bean mass from position a to b or vice versa (Fig 1a). The two parts of the boxes a and b were separated by moveable wooden planks. The fermentations lasted 4 to 8 days and the end of each individual fermentation was determined by a cut-test with 25 beans randomly selected from different parts of the box. When ≥ 65% of the beans were well-fermented, i.e. the cotyledons had turned brownish and exhibited a wrinkled structure, the process was stopped by heaping the pulp-bean mass on a drying tray with a layer thickness of about 12 cm and leaving it to rest overnight in the fermentation building. A predrying process was initiated the following day by reducing the layer thickness to 5–6 cm and exposing the pulp-bean mass to the sun and turning it every 30 min. During the predrying phase, i.e. drying days 1, 2, and 3, the beans were exposed to direct sunlight for 2 h, 4 h, and 6 h, respectively. The main drying phase was initiated on drying day 4, during which the beans were exposed to the sun for 8 h daily until a water content of 6–7% was reached, which was measured using a portable coffee moisture tester (AgraTronix Corporate, Ohio, USA).

Samples were collected every 24 h during the fermentation process while mixing the pulp-bean mass, the first sample being taken after inoculation of the beans at 0 h and the last sample on the final day of fermentation, by combining equal amounts from nine positions in the box as described by Romanens et al. (2018) [4]. During drying, samples were collected by combining equal amounts of drying cocoa beans from the middle, one side, and one corner of the drying platform. Samples were collected daily during the predrying stage, i.e. on drying days 1, 2, and 3, and every second day during the main drying phase, i.e. on drying days 5, 7, 9, etc. and on the final day for fully dried beans. The samples for microbial count, pH, and pulp content analysis were processed within 2–3 hours on the day of sampling when possible, while samples for polyphenol analyses were stabilized with a 5 g/l CaCl_2_ solution in a sample-to-solvent ratio of 1:7 according to Romanens et al. (2018) [4]. These samples, together with samples for microbial substrate and metabolite analysis, were transported to Switzerland on ice and stored at -20 °C, while samples for sensorial analyses were stored and transported at room temperature. A reference sample of dried cocoa beans that were commercially fermented at Chocolats Halba Honduras (San Pedro Sula, Cortés, Honduras) was obtained from Chocolats Halba (Wallisellen, Switzerland) for sensory and substrate and metabolite analysis.

### Determination of microbial counts

Concentrations of the dominant microbial groups on the surface of cocoa beans were determined using various growth media as described by Romanens et al. (2018) [4]. Plate count agar (PCA) (Fluka, St. Louis, USA) was used to quantify aerobic mesophilic germs, Yeast Extract Glucose Chloramphenicol (YGC) agar (Biolife, Milan, Italy) for yeasts, De Man-Rogosa-Sharpe (MRS) agar (Biokar Diagnostics, Beauvais, France) for LAB, and Ethanol Glucose Mannitol (EGM) agar [21] and Yeast Peptone Mannitol (YPM) for AAB. To suppress eukaryotic cell growth, 0.1 g/l cycloheximide (Sigma-Aldrich, St. Louis, USA) was added to MRS, EGM, and YPM agar, while EGM and YPM agar were additionally supplemented with 0.05 g/l penicillin (Ulticilin de Honduras SA, Honduras) to inhibit the growth of remaining Gram-positive bacteria. Serial dilutions were prepared, starting with 10 g cocoa pulp bean mass that was mixed with 9 times its own weight of dilution solution and treated for 1 min at 180 rpm using a Stomacher 400 (Seward, Worthington, UK) and appropriate dilutions were applied on the respective growth medium. PC agar was incubated aerobically at 30 °C for 3 days, MRS agar anaerobically at 37 °C for 3 days, and EGM, YPM, and YGC aerobically at room temperature (approx. 20–26 °C) for 4–5 days for AAB and 3 days for yeasts, respectively. One to ten colonies from an appropriate dilution with different morphological appearances were selected for confirmation tests. Presumptive yeasts from YGC were confirmed microscopically as yeasts, bacterial isolates from MRS with Gram-positive and catalase-negative behaviour as LAB, and bacterial isolates from EGM and YPM with Gram-negative and catalase-positive behaviour as AAB.

### Measurement of pulp content, pH, and temperature

The content of pulp was determined as single measurements using the method previously described [4] and expressed as a percentage of the pulp weight compared to the whole bean including the adherent pulp. To determine pulp pH, 20 g of pulp-bean mass was manually mixed with 9 times its own weight of dH_2_O for 1 min and pH was measured in the pulp-water mix using a portable pH-meter (VWR International, Pennsylvania, USA). Similarly, the cotyledon pH was determined in a cotyledon-water mix produced by homogenizing 20 g of cotyledons after removing pulp and testa manually with 9 times the weight of dH_2_O for 2 min using a hand blender (Bamix AG, Mettlen, Switzerland). The temperature of the fermentation mass was recorded every 15 min with nine temperature sensors (DS18B20, Maxim Integrated, California, US) that were placed in different spots in the fermentation box as described [4].

### Determination of microbial substrates and metabolites

To determine microbial substrates and metabolites in pulp and cotyledon, water extracts were prepared from pulp samples from fermentation and predrying and from cotyledon samples collected during fermentation and the whole drying process including dried beans, according to the method of Camu et al. (2007) [22] with some modifications. Due to an increasingly lower water content in the pulp during the late fermentation and the predrying stages, 10 g rather than 20 g of pulp were used from the predrying phase and the amount of MilliQ water applied during the first extraction step was increased from 80 ml to 130 ml for pulp samples collected on fermentation day 4 until the end of predrying. The sample-water mixes were homogenized using a hand blender (Bamix AG) for 2 min in the case of pulp samples and 1 min for cotyledon samples, with the exception that cotyledon samples collected from the main drying phase, i.e. from drying day 5 until the end of drying, were treated for 1.5 min. The combined supernatants were diluted with MilliQ water 1:1 for pulp samples from fermentation days 0 to 3 and 1:0.5 for pulp samples from fermentation day 4 until the last day of fermentation, whereas no additional dilution step was applied to pulp-sample supernatants from the predrying phase and to cotyledon-sample supernatants. Subsequently, an extra centrifugation step was completed for all pulp sample extracts at 17’000 ×g for 15 min at 4 °C. The resulting supernatants were filtered through 0.2-μm filters (Sartorius AG, Goettingen, Germany), whereas combined supernatants from cotyledon samples were treated with 0.45-μm filters (Sartorius AG). The filtrates were kept frozen at -20 °C. Before measurement, extracts from cotyledon samples were filtered through 0.2-μm filters to eliminate precipitates formed during the freezing step. Sucrose, glucose, fructose, citric acid, ethanol, lactic acid, acetic acid, gluconic acid, and mannitol were measured in water extracts using high-performance liquid chromatography with refractive index detector HPLC-RI (Hitachi, Merck KGaA, Darmstadt, Germany) in duplicate as described by Romanens et al. (2018) [4].

### Determination of polyphenols and alkaloids

Total phenolic contents (TPC) and concentrations of the polyphenols, gallic acid, (+)-catechin, (–)-epicatechin, proanthocyanidin B2, proanthocyanidin C1, cinnamtannin A2, quercetin-3-*O*-galactoside, quercetin-3-*O*-glucoside, quercetin-3-*O*-arabinoside, cyanidin-3-*O*-galactoside, and cyanidin-3-*O*-arabinoside, and of the alkaloids, caffeine and theobromine, were analyzed in samples from fermentation days 0 and 2, the last fermentation day, the last predrying day, i.e. drying day 3, and fully dried beans. The stabilized cotyledon samples were defatted and extracted with a 50% acetone-water solution. The total phenolic contents (TPC) of these extracts were measured using Folin-Ciocalteu assay, whereas alkaloids and single polyphenols were measured with liquid chromatography coupled to diode array detection and electrospray ionization spectrometry (LC-DAD-ESI/MS) as described by Pedan et al. (2016) [23].

### Cut test and sensory analysis of dried cocoa beans

The quality of dried beans resulting from each fermentation was assessed by a cut test with 300 dried cocoa beans and by tasting cocoa liquors as described [4]. The cocoa liquors were prepared and evaluated sensorially by a trained panel of seven at Chocolats Halba (Wallisellen, Switzerland) in August 2017.

## Results

### Impact of fermentation-type on fermentation- and drying-time and heat generation

Fermentations with co-culture A (F-A) lasted 6, 5, and 8 days, fermentations with co-culture B (F-B) 5, 7, and 8 days, and spontaneous control fermentations (F-S) 4, 7, and 8 days during IF, IIF, and IIIF, respectively (Fig 1b). The drying of the beans lasted 9–10 days during IF, 13–15 days during IIF, and 10 days during IIIF.

**Fig 2:**
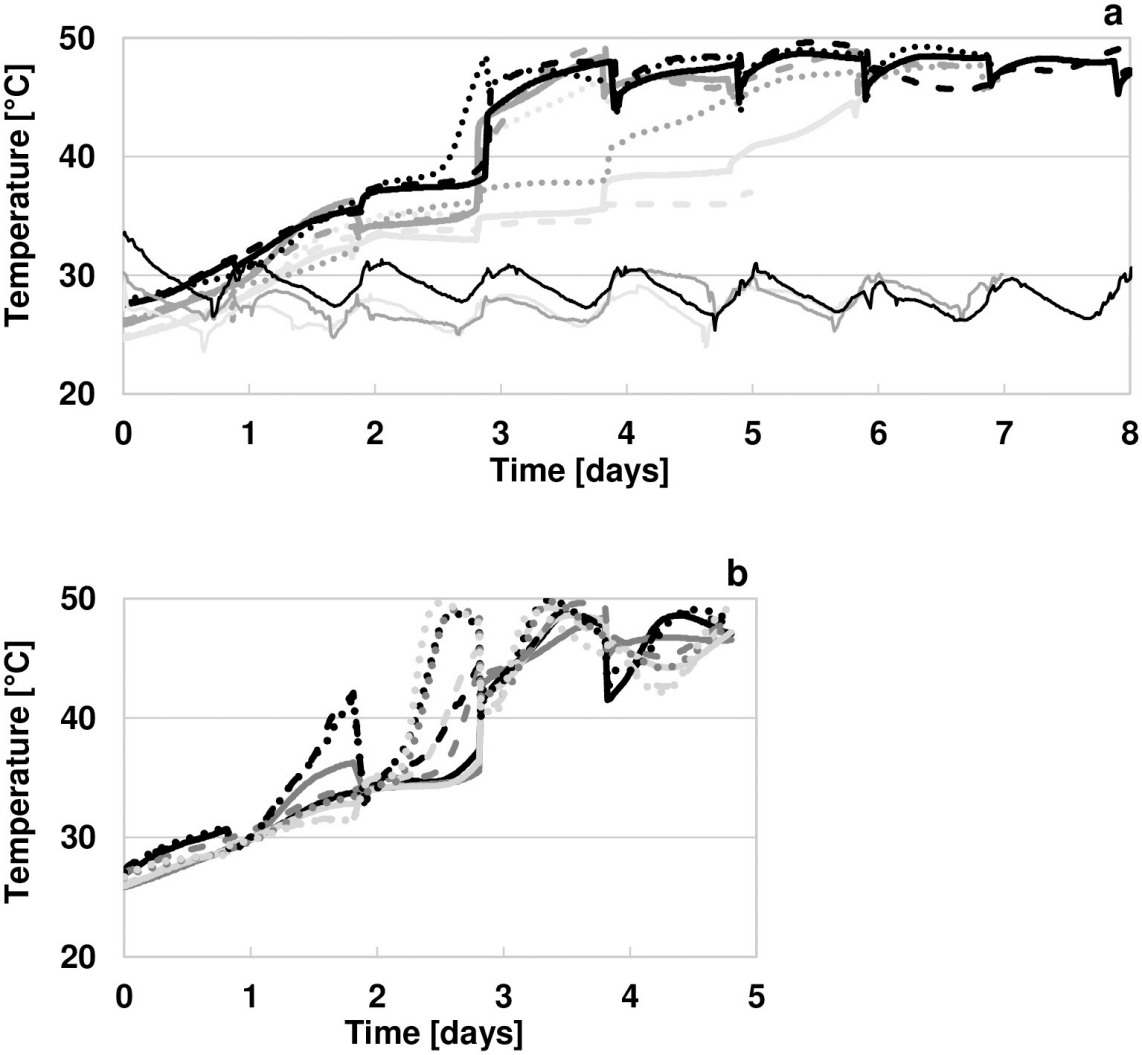
Temperatures in the fermenting pulp-bean mass of inoculated and spontaneous fermentations (a) and temperature distribution in the fermentation mass of IIF-A, representative for the nine studied fermentations (b). In part (a), temperatures measured in the centre of the box are shown for fermentation IF-A (bold bright grey line), IF-B (dashed bright grey line), IF-S (dotted bright grey line), IIF-A (bold dark grey line), IIF-B (dashed dark grey line), IIF-S (dotted dark grey line), IIIF-A (bold black line), IIIF-B (dashed black line), and IIIF-S (dotted black line). Ambient temperatures were measured in the fermentation building during run I (thin bright grey line), run II (thin dark grey line), and run III (thin black line). Part (b) shows temperatures measured at nine positions in fermentation box IIA: bottom-centre (solid bright grey line), bottom-side (dashed bright grey line), bottom-corner (dotted bright grey line), centre-centre (solid dark grey line), centre-side (dashed dark grey line), centre-corner (dotted dark grey line), top-centre (solid black line), top-side (dashed black line), and top-corner (dotted black line). 1–2 values deviated due to cold air being mixed with the pulp-bean mass and these were deleted to provide a clearer indication of temperature trends.

Ambient temperatures peaked during the day and were lowest during IF, at 24–30 °C, slightly higher during IIF at 25–31 °C, and highest during IIIF at 25–34 °C (Figs 1b and 2). The fermentation mass temperature in the centre of the fermentation boxes started at 25–28 °C and increased gradually to maximum temperatures of 45–50 °C, except for fermentation IF-B, which reached only 37 °C. Temperatures in the centre of the fermentation mass at the highest levels of 45–50 °C were measured during 5 days in IIIF, during 4 days in IIF-B, during 2 days in IIF-A and IIF-S, during 0.5 days in fermentation IF-S, and during the last hour in IF-A (Fig 1b). During IF and IIIF, 8–12 °C lower temperatures were recorded in inoculated compared to spontaneous control fermentations and during all three fermentation runs, boxes placed in position 3 close to the door, i.e. IF-B, IIF-S, and IIIF-A, showed up to 9–12 °C cooler temperatures than boxes placed in positions 1 and 2 in the back of the building (Figs 1 and 2a). The heat inside the fermentation box was not distributed homogeneously as exemplified for IIF-A in Fig 2b. After a smooth temperature increase in all areas of the box on day 1, temperatures rose primarily in the upper regions during 1–2 days, followed by 1–2 days with sharp temperature increases in the corner positions as well as in the side-bottom position. From fermentation day 4 on and especially during IIF and IIIF for whichthe gaps in the boxes had been closed with wooden slats, the temperature distribution was more homogenous.

### pH development and pulp content

The content of 28–36% of pulp in fresh cocoa-pulp bean mass decreased continuously to 12–19% towards the end of the fermentations and further to 2–7% by the end of predrying, i.e. drying day 3 (Table 1). The initial acidic pulp pH of 3.8–3.9 increased to 4.3–4.4 in IF and IIF and 4.5–4.7 IIIF on the last fermentation day (Table 1) due to microbial metabolization of citric acid (S1 Table). At the same time, the cotyledon pH decreased in all fermentations from 6.6–6.7 in fresh beans to minimal levels of 4.4–4.9. During IIIF, the cotyledon pH subsequently increased during the last 2–3 fermentation days to 4.7 on day 8, the final day of fermentation. During predrying, a sharp increase of pulp pH to 6.6–7.7 was observed, before pH values dropped to final levels of 5.7–6.7 during the main drying phase, whereas cotyledon pH increased gradually to 5.0–5.2 in dried beans after IF and IIF and higher levels of 5.5–5.6 after IIIF.

**Table 1:** Pulp content, pulp pH, and cotyledon pH during inoculated and spontaneous fermentation and drying processes of IF, IIF, and IIIF.

Treatment	Box position	Fermentation				Drying			
		Day 0	Min.	Day	End	Day 3	Max.	Day	End
		**Pulp content [%; w/w]**							
IF-A	2	34	-	-	19	6	-	-	n.d.
IF-B	3	33	-	-	15	2	-	-	n.d.
IF-S	1	28	-	-	15	4	-	-	n.d.
IIF-A	1	34	-	-	15	5	-	-	n.d.
IIF-B	2	36	-	-	13	6	-	-	n.d.
IIF-S	3	35	-	-	16	7	-	-	n.d.
IIIF-A	3	30	-	-	12	5	-	-	n.d.
IIIF-B	1	32	-	-	12	6	-	-	n.d.
IIIF-S	2	31	-	-	13	7	-	-	n.d.
		**pH pulp**							
IF-A	2	3.9	-	-	4.3	-	7.2	2	5.7
IF-B	3	3.9	-	-	4.3	-	7.7	2	6.5
IF-S	1	3.8	-	-	4.3	-	7.7	3	6.2
IIF-A	1	3.8	-	-	4.3	-	7.2	2	6.5
IIF-B	2	3.9	-	-	4.4	-	6.6	11	6.5
IIF-S	3	3.9	-	-	4.4	-	6.9	2	6.4
IIIF-A	3	3.8	-	-	4.7	-	7.2	2	6.7
IIIF-B	1	3.8	-	-	4.5	-	7.0	2	6.5
IIIF-S	2	3.8	-	-	4.6	-	7.4	2	6.4
		**pH cotyledon**							
IF-A	2	6.6	4.4	6	4.4	4.7	-	-	5.0
IF-B	3	6.6	4.9	5	4.9	4.9	-	-	5.0
IF-S	1	6.6	4.8	4	4.8	5.0	-	-	5.1
IIF-A	1	6.6	4.5	5	4.5	4.9	-	-	5.2
IIF-B	2	6.7	4.4	6	4.5	5.0	-	-	5.2
IIF-S	3	6.7	4.4	7	4.4	4.8	-	-	5.0
IIIF-A	3	6.7	4.5	6	4.7	5.3	-	-	5.5
IIIF-B	1	6.7	4.5	5	4.7	5.2	-	-	5.5
IIIF-S	2	6.7	4.4	6	4.7	5.3	-	-	5.6

Min. = minimal concentration during fermentation; Max. = maximal concentration during drying; Day = day on which maximal or minimal concentration was detected;— = not applicable; n.d. = not determined.

### Microbial succession in the fermentation and drying process

The courses of total aerobic germs, yeasts, LAB, and AAB were analyzed throughout fermentation and drying processes (Table 2). Starting at concentrations of 4.7–6.9 log CFU/g in fresh pulp-bean mass, microbial concentrations increased in the first four fermentation days to 7.9–8.9 log CFU/g of total aerobic germs, 6.2–7.5 log CFU/g of yeasts, with 0.5–0.9 log CFU/g higher yeast counts in F-A than in F-B, 7.9–9.5 log CFU/g of LAB, and 6.3–7.6 log CFU/g of AAB. During IIIF, microbial counts reached their maxima up to 3 days earlier than in IF and IIF. Subsequently, with increasing temperatures, microbial counts dropped to 3.0–7.4 log CFU/g, except for yeasts that dropped below detection limit in most fermentations. Minimal counts measured during fermentation were 0.1–3.1 log CFU/g higher when the box was placed in position 3 compared to positions 1 and 2, except for AAB counts that were in a similar range independent of the position of the fermentation box. In fermentations that lasted longer than 6 days, i.e. IIF-B, IIF-S, and IIIF, microbial concentrations increased to 6.0–6.8 log CFU/g for total aerobic mesophilic germs, 2.0–2.8 log CFU/g of yeasts, 6.6–8.0 log CFU/g of LAB, and 4.7–6.6 log CFU/g of AAB on the final fermentation day.

**Table 2:** Cell counts of total aerobic germs, yeasts, LAB, and AAB during inoculated and spontaneous fermentation and drying processes of IF, IIF, and IIIF.

Treatment	Box position	Fermentation						Drying		
		Day 0	Max.	Day	Min.^b^	Day	End	Max.	Day	End
		**Total aerobic germs [log CFU/g]**								
IF-A	2	6.7	8.9	3	5.2	6	5.2	6.9	2	6.4
IF-B	3	6.8	7.9	1	7.1	5	7.1	7.5	3	5.7
IF-S	1	6.8	8.8	2	4.5	4	4.5	7.8	3	6.5
IIF-A	1	6.9	8.4	2	5.0	5	5.0	8.4	3	6.4
IIF-B	2	6.7	8.2	2	4.6	5	6.1	7.9	3	5.9
IIF-S	3	6.8	8.1	2	4.9	6	6.0	7.7	3	6.5
IIIF-A	3	6.7	8.3	1	5.1	6	6.8	8.6	5	7.7
IIIF-B	1	6.1	8.3	1	4.7	6	6.5	8.5	10	8.5
IIIF-S	2	5.3	8.1	2	4.7	5	6.7	8.8	3	7.7
		**Yeasts [log CFU/g]**								
IF-A	2	6.0	7.4	4	<	6	<	3.8	3	<
IF-B	3	6.1	6.8	2	6.1	5	6.1	4.8	2	3.5
IF-S	1	6.6	7.2	2	3.0	4	3.0	5.1	3	3.7
IIF-A	1	6.1	7.5	3	<	5	<	4.4	3	<
IIF-B	2	6.2	6.6	2	<	5	2.6	4.9	2	<
IIF-S	3	6.1	6.7	2	<	6	2.6	5.0	3	3.2
IIIF-A	3	6.0	6.7	1	<	6	2.8	5.3	1	4.2
IIIF-B	1	5.7	6.2	1	<	4	2.5	6.0	3	4.4
IIIF-S	2	5.4	6.7	2	<	4	2.0	5.3	5	3.7
		**Lactic acid bacteria [log CFU/g]**								
IF-A	2	5.9	9.5	1	7.3	4	8.6	7.2	1	5.5
IF-B	3	6.3	8.3	1	7.4	3	7.4	7.8	3	2.0
IF-S	1	5.9	8.0	1	5.8	4	5.8	8.6	3	7.3
IIF-A	1	6.1	8.2	2	4.5	4	5.2	8.8	3	7.1
IIF-B	2	6.3	8.4	2	3.6	5	7.3	8.7	2	7.0
IIF-S	3	5.6	7.9	2	6.5	5	6.6	8.7	5	7.1
IIIF-A	3	6.7	8.4	1	5.3	4	8.0	8.6	1	7.6
IIIF-B	1	5.3	8.3	1	5.2	4	7.7	8.6	1	7.8
IIIF-S	2	<	8.6	2	4.8	5	7.4	8.9	3	7.8
		**Acetic acid bacteria [log CFU/g]**								
IF-A	2	6.2	7.1	3	5.0	6	5.0	7.4	1	5.1
IF-B	3	6.6	6.7	3	6.4	5	6.4	8.2	2	4.8
IF-S	1	6.6	7.6	3	4.4	4	4.4	7.8	3	5.5
IIF-A	1	6.1	6.4	2	4.3	5	4.3	7.7	2	5.0
IIF-B	2	6.1	6.6	7	4.8	6	6.6	7.9	1	4.8
IIF-S	3	6.3	6.8	2	3.6	6	5.9	7.2^a^	3	3.1
IIIF-A	3	5.9	6.6	3	5.3	6	6.2	6.8	1	<
IIIF-B	1	4.9	6.3	2	3.6	6	4.7	6.9	1	<
IIIF-S	2	4.7	6.9	2	5.4^a^	3	5.7^a^	6.3	1	<

Detection limits were 2 log CFU/g for yeasts and LAB and 2.7 log CFU/g for total aerobic germs and AAB. Max. = maximal concentration during fermentation or drying; Min. = minimal concentration during fermentation; Day = day on which the maximal or minimal value was detected: if the maximal or minimal value was recorded on more than one day, the first day with the same maximal or minimal value was listed; < = below detection limit.

^a^Cell counts on one of two media below detection limit.

^b^When minimal cell counts were measured on day 0 for LAB or day 1 for AAB, the second-lowest concentration was listed.

During drying, microbial counts reached a second peak primarily during the predrying phase, with 6.9–8.8 log CFU/g of total aerobic germs, 3.8–6.0 log CFU/g of yeasts, 8.6–8.9 log CFU/g of LAB, and 6.3–8.2 log CFU/g of AAB (Table 2). Microbial counts decreased slightly to final levels on dried beans of 5.7–8.5 log CFU/g of total aerobic germs, ≤ 4.4 log CFU/g of yeasts, 2.0–7.8 log CFU/g of LAB, and ≤ 3.1–5.5 log CFU/g of AAB. Highest total mesophilic aerobic, yeast, and LAB counts were detected during drying of IIIF performed in April when the ambient temperature was highest, followed by IIF, and lowest counts on drying beans were observed in IF when lowest ambient temperatures were measured, while the contrary was observed for AAB, with lower counts during drying in IIIF followed by IF and IIF.

Colony morphologies of LAB on MRS agar and yeasts on YGC agar were monitored (data not shown). During the first 3–6 days of F-A and F-B, colonies with a similar macro- and microscopical appearance to *Lb*. *fermentum* strains M017 and 223 dominated, suggesting that the inoculated *Lb*. *fermentum* strains M017 and 223 dominated the LAB microbiota during F-A and F-B, respectively, whereas during F-S, a diversity of colony morphologies was observed. For yeasts, no differences in colony morphologies were observed between inoculated and spontaneous fermentations, suggesting that the inoculated *S*. *cerevisiae* strain H290 did not or not completely suppress indigenous yeast flora.

### Changes of microbial substrates and metabolites during fermentation and drying

**Fig 3:**
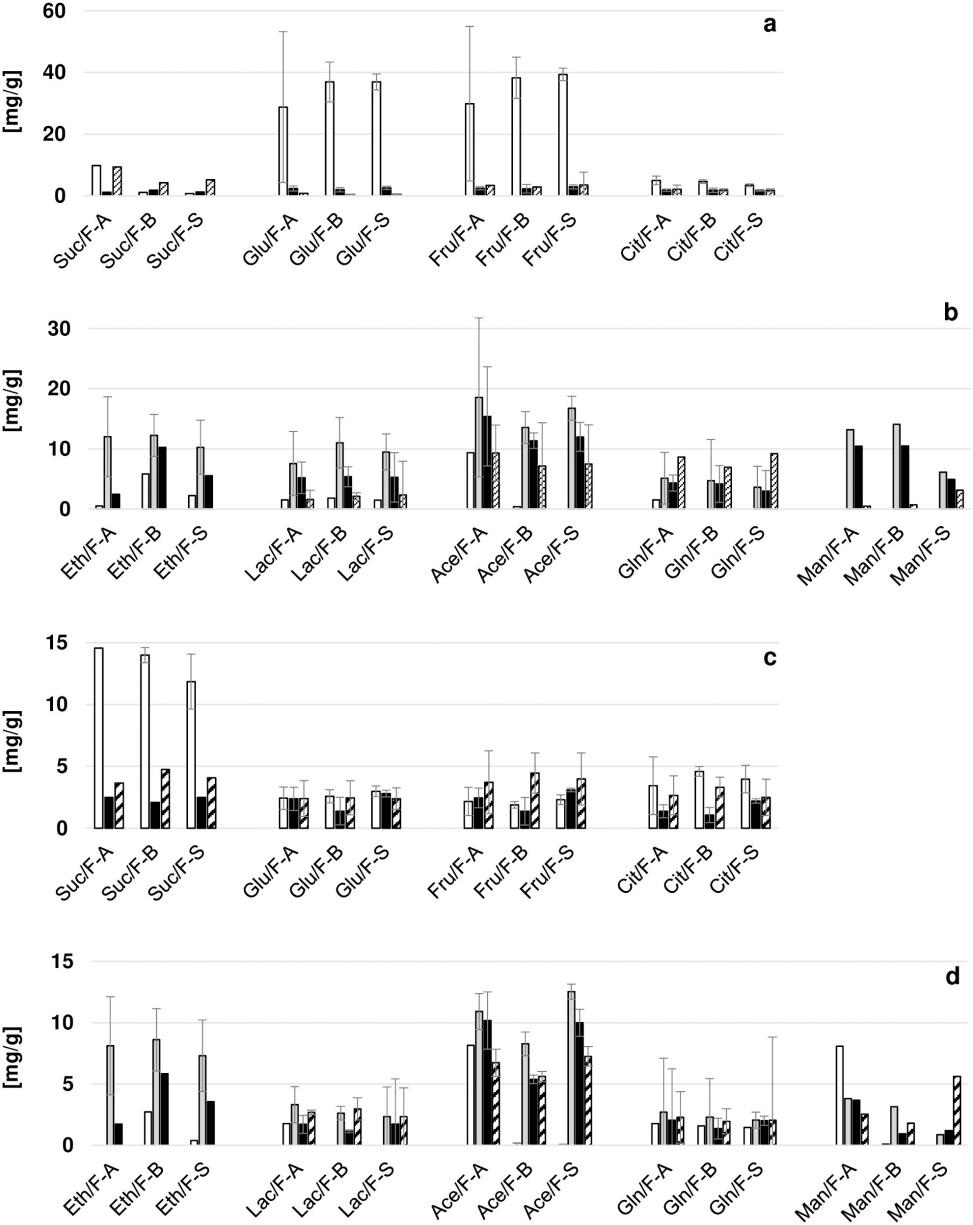
Concentrations of microbial substrates in pulp (a) and cotyledons (c) and of metabolites in pulp (b) and cotyledons (d) during F-A, F-B, and F-S. The concentrations of substrates and metabolites are shown for fermentation day 0 (white), for the final fermentation day (black), and for dried beans (stripes) and the maximum values of metabolites measured during fermentation are shown (grey). Mean values were calculated from the three replicate fermentations performed during IF, IIF, and IIIF. Suc = sucrose; Glu = glucose; Fru = fructose; Cit = citric acid; Eth = ethanol; Lac = lactic acid; Ace = acetic acid; Gln = gluconic acid; Man = mannitol.

The course of substrates and metabolites measured in samples from fermentation and drying were comparable for the nine fermentations, except for IIIF-A having a different sugar composition and higher metabolite concentrations in fresh pulp bean mass (Fig 3; S1 Table). The main sugars in fresh pulp were glucose and fructose with concentrations of 29.7–44.9 mg/g, while the sucrose level was low or below detection limit. Pulp glucose and fructose were almost depleted after 2–3 days due to microbial activity. Contrary to the pulp, sucrose was the main sugar in fresh cotyledons at 10.2–17.4 mg/g and decreased during fermentation to minimal values of ≤ 2.8 mg/g on days 4 to 7, while no relevant changes were observed for glucose and fructose levels in cotyledons. In fully dried beans, levels of glucose were at 2.6–3.3 mg/g after IF and IIF and at 0.7–1.4 mg/g after IIIF and levels of fructose at 4.5–5.2 mg/g after IF and IIF, and at 0.9–2.6 mg/g after IIIF. Concentrations of sucrose in dried beans were at 3.6–4.1 mg/g after IF, while no sucrose was detected after IIF and IIIF. Initial levels of 2.9–6.6 mg/g of citric acid in pulp and cotyledons of fresh pulp-bean mass decreased to minimal levels of 0.2–0.4 mg/g within 1–3 days for pulp citric acid and to 0.4–2.1 mg/g within 4–8 days for cotyledon citric acid (Fig 3; S1 Table). In cotyledons of dried beans, levels of citric acid of 3.0–4.2 mg/g were measured after IF and IIF, and of 0.8–2.6 mg/g after IIIF.

From the sugars and citric acid in the pulp, microorganisms produced ethanol, organic acids, and mannitol (Fig 3; S2 Table). Maximal ethanol levels in the pulp of 8.9–18.2 mg/g were measured in IF and IIF and slightly lower concentrations of 5.0–8.6 mg/g during IIIF. Parts of the ethanol diffused into the beans, where ethanol reached highest maximal levels during IF with 10.3–12.0 mg/g, slightly lower levels during IIF with 7.2–9.4 mg/g, and lowest levels during IIIF with 4.0–5.8 mg/g. Up to 3.9 mg/g higher maximal levels of ethanol were measured in pulp and cotyledons of F-A and F-B compared to F-S. The ethanol levels decreased throughout fermentation and/or drying and reached levels below detection limit in pulp on the final predrying day and in cotyledons of dried beans. At the same time when maximal ethanol concentrations occurred, i.e. after 2–3 days, maximal levels of lactic acid were found in the pulp at 5.9–17.1 mg/g, whereas the pulp acetic acid peak occurred only after 4–7 days at 6.0–31.5 mg/g (Fig 3; S2 Table). Up to 6 times lower pulp acetic acid levels were found at the end of 8-day fermentations, i.e. IIIF, compared to pulp acetic acid levels at the end of shorter fermentations of 4–7 days, i.e. IF and IIF. In the cotyledons, lactic acid reached peaks of 1.7–6.2 mg/g on days 2–6 and, due to a fast diffusion from the pulp into the beans, the volatile acetic acid reached maximal cotyledon levels of 5.7–14.1 mg/g on days 3–6. The lactic acid concentration subsequently decreased to 0.9–3.9 mg/g in the pulp on drying day 3, the last day of predrying, while it remained stable in the cotyledons, which resulted in final levels of 1.7–3.4 mg/g in dried beans. A sharp decrease of acetic acid concentration was observed mainly during the first predrying day of IF and IIF and in the last 3–6 fermentation days of IIIF (data not shown). The acetic acid levels in dried beans were at 6.2–9.1 mg/g after IF and IIF and at lower levels of 2.5–6.3 mg/g after IIIF. In F-A, acetic acid levels were up to 2 times higher in the pulp of fully fermented beans and cotyledons of dried beans compared to F-B. Independent of inoculation with anti-fungal cultures, acetic acid concentration in dried bean cotyledons were 3–10 times higher after the nine studied fermentations than in the sample of commercially fermented reference cocoa beans. In fresh pulp-bean mass, gluconic acid was only detected in cotyledons, at levels of 1.3–1.9 mg/g (Fig 3; S2 Table). Gluconic acid in the pulp reached maximal levels of 5.0–14.2 mg/g during predrying of IF and IIF and 3.4–8.2 mg/g on fermentation days 6 or 7 during IIIF. In the cotyledons, gluconic acid levels only increased slightly during fermentation and/or drying, resulting in cotyledon gluconic acid concentrations of 1.2–2.8 mg/g in dried beans. Maximal mannitol concentrations during fermentation were up to 10 times higher in F-A and F-B at 8.6–21.8 mg/g in pulps and 0.9–8.9 mg/g in cotyledons than during F-S at 2.5–9.5 mg/g in pulps and 0.2–1.5 mg/g in cotyledons, respectively (Fig 3; S2 Table). Pulp mannitol decreased subsequently to levels of ≤ 3.1 mg/g on the final day of predrying, whereas final mannitol levels in dried beans were detected at up to 10.4 mg/g.

### Alkaloid and polyphenolic contents of cotyledon samples

**Fig 4:**
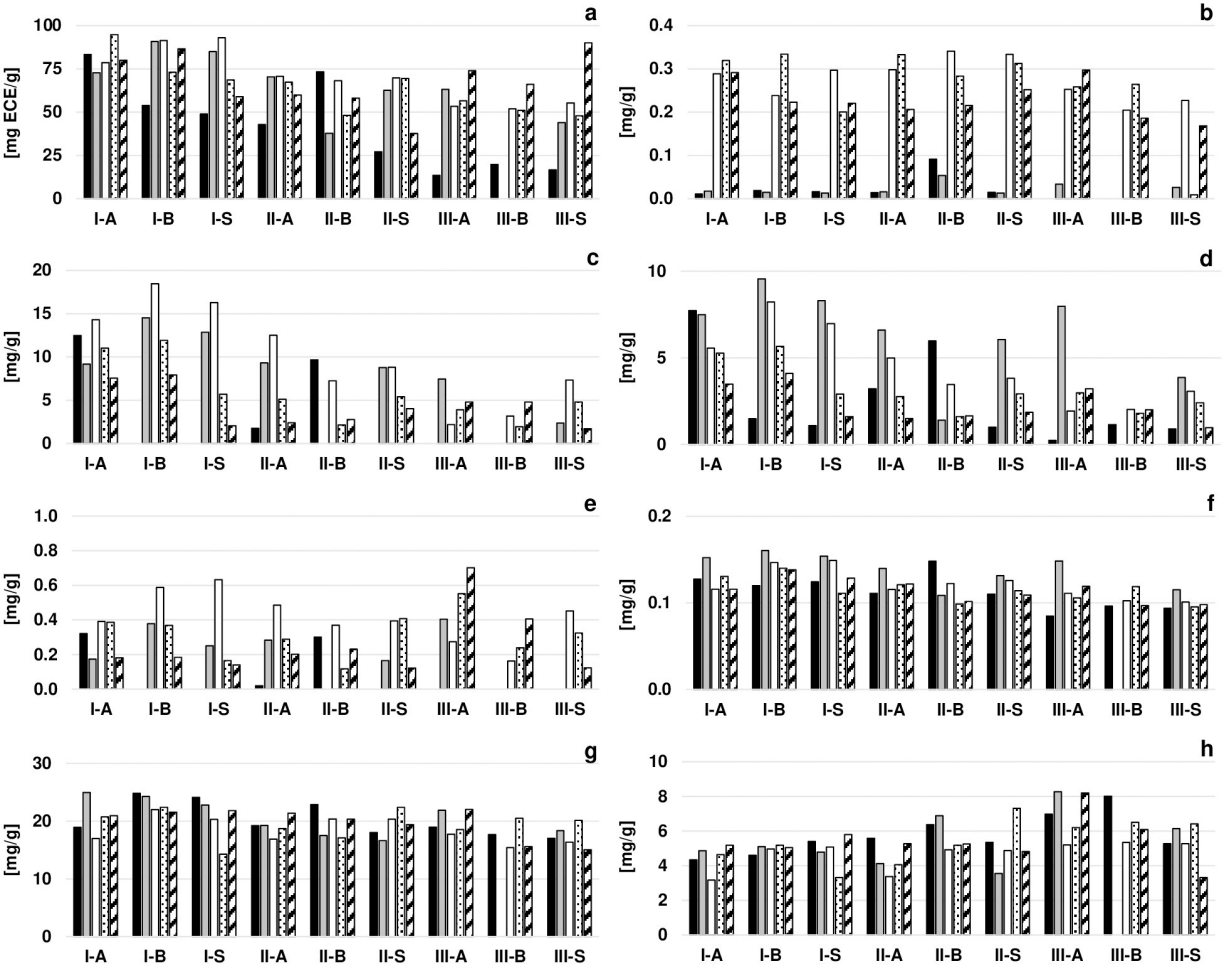
Total phenolic content (TPC) in epicatechin equivalents (ECE; a) and contents of gallic acid (b), (–)-epicatechin (c), proanthocyanidin C1 (d), (+)-catechin (e), quercetin-3-*O*-glucoside (f), theobromine (g), and caffeine (h) during inoculated and spontaneous fermentation and drying processes. Values are expressed in milligrams per gram of non-fatty dry matter of cotyledons on fermentation day 0 (black), fermentation day 2 (grey), the last fermentation day (white), drying day 3 (dotted), and for dried beans (striped).

The alkaloids theobromine and caffeine and relevant polyphenolic compounds of cocoa beans were determined at different points during fermentation and drying and courses of total phenolic content (TPC), and contents of gallic acid, (–)-epicatechin, proanthocyanidin C1, (+)-catechin, quercetin-3-*O*-glucoside, theobromine, and caffeine are exemplified for the 13 substances measured in Fig 4. TPC, expressed as (–)-epicatechin equivalents (ECE) per gram of fat-free dry matter of cotyledons, ranged between 13.5 mg ECE/g and 83.2 mg ECE/g in fresh bean cotyledons (Fig 4a). In most fermentations, an increase of TPC was observed to 52.0–93.0 mg ECE/g on the last fermentation day. During drying, concentrations slightly decreased in IF and IIF to 37.6–86.5 mg ECE/g and increased in IIIF to 66.1–90.0 mg ECE/g in dried beans. The monomeric polyphenolic compounds, (–)-epicatechin (Fig 4c) and (+)-catechin (Fig 4e), increased throughout the fermentation processes to concentrations of 2.2–18.5 mg/g and 0.2–0.6 mg/g, respectively, on the final fermentation day. Subsequently, their concentrations decreased during the drying processes to 1.7–7.9 mg/g of (-)-epicatechin and 0.1–0.2 mg/g of (+)-catechin in dried beans. Proanthocyanidin B2, proanthocyanidin C1 (Fig 4d), cinnamtannin A2, i.e. polymers of epicatechin, increased to 1.8–8.3 mg/g in fully fermented beans and decreased subsequently during drying to concentrations of 0.8–4.8 mg/g in dried beans. Gallic acid (Fig 4b) was produced primarily at the end of the fermentation process at concentrations of 0.20–0.34 mg/g and the concentration remained stable during drying. The concentrations of glycosidic quercetin compounds quercetin-3-*O*-galactoside and quercetin-3-*O*-glucoside (Fig 4f) ranged between 0.07 and 0.16 mg/g during fermentation and drying, with slightly higher values on fermentation days 0 and 2 than during the remaining process, while quercetin-3-*O*-arabinoside at 0.08 mg/g was only measured in a few fermentations on days 0 and/or 2. The major anthocyanins, cyanidin-3-*O*-galactoside and cyanidin-3-*O*-arabinoside, were measured at concentrations of 0.05–0.72 mg/g on fermentation days 0 and 2 and were below detection limit on the last fermentation day except for 0.07 mg/g of cyanidin-3-*O*-arabinoside at the end of fermentation IB. The concentrations of theobromine (Fig 4g) and caffeine (Fig 4h) were measured at 4.3–25.0 mg/g and 3.2–8.3 mg/g, respectively, and no influence of fermentation or drying was observed.

### Fermentation degree and sensory profile of dried beans

**Fig 5:**
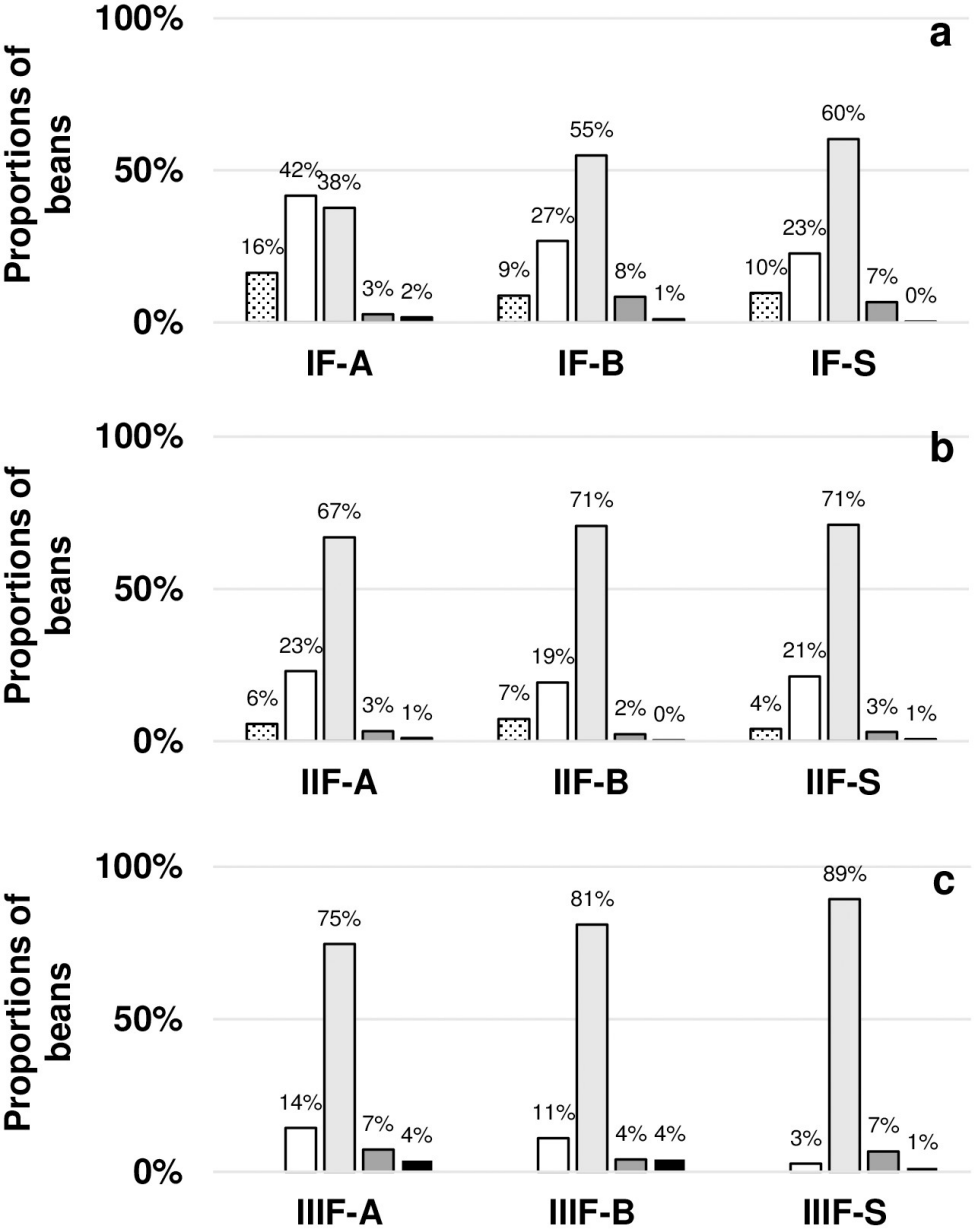
Evaluation of dried beans revealed by a cut test with 300 beans after inoculated and spontaneous processes during IF (a), IIF (b), and IIIF (c). Bean proportions are expressed in percentage classified as violet (black dots on white background), slightly violet (white), fully fermented (light grey), over fermented (dark grey), and mouldy (black).

In a cut test, 300 dried beans from each individual fermentation were evaluated based on their fermentation degree and other quality attributes (Fig 5). None of the fermentations achieved the requirements for quality A cocoa beans defined by the chocolate producing company Chocolats Halba (Wallisellen, Switzerland). The requirement of ≥ 75% of well-fermented beans was met for IIIF, at 75–89%, but not for IF and IIF at 38–60% and 67–71%, respectively. The highest proportions of well-fermented beans were detected after F-S, followed by F-B, and lowest proportions among beans resulting from F-A processes. The proportions of over-fermented beans were above the threshold of ≤ 3% for IF-B, IF-S, and IIIF. Mouldy beans were below the limit of ≤ 3% at 0–2% after all fermentations except for IIIF-A and IIIF-B at 4% of mould-infested beans. No slaty beans were detected in either of the dried bean samples.

**Fig 6:**
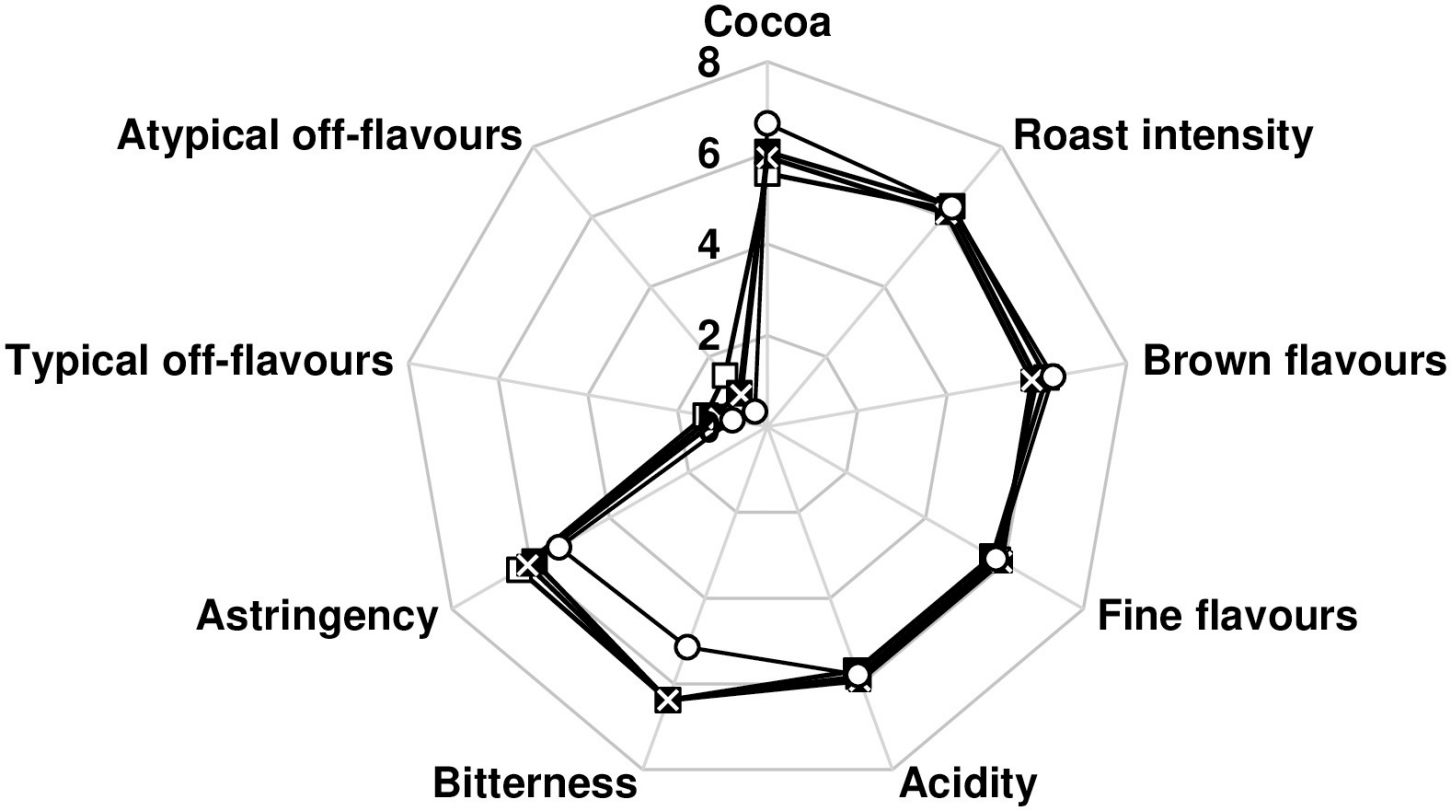
Sensory profiles of cocoa liquors produced from dried beans after F-A (□), F-B (■), and F-S (×) and from reference dried beans (○).

The flavour attributes of cocoa liquors were rated on a scale from 0 to 10 (Fig 6, S3 Table) to determine the sensory profile of dried bean samples. The inoculation with culture B, compared to culture A, led to 0.2–0.6 and ≤ 1.1 units lower astringency and typical and atypical off-flavours, respectively, and to 0.2–0.9 and 0.5–0.7 units higher cocoa flavours and acidity, respectively. No differences between F-A, F-B, and F-S were noted for the flavour attributes with roast intensity ranging from 5.9 to 6.6, fine flavours from 5.5 to 6.1, brown flavours from 5.4 to 6.4, and bitterness from 6.1 to 6.8. Compared to the commercially fermented reference cocoa bean sample, the nine fermentations studied were rated 1.0–1.7 units higher in bitterness, 0.3–1.2 units higher in astringency, and 0.2–2.0 units higher in typical and atypical off flavours.

## Discussion

The primary function of anti-fungal cultures is to suppress filamentous fungi and, unlike for starter cultures, not to stabilize or improve the fermentation process. However, it is of critical importance that the anti-fungal cultures do not negatively influence the quality of the end product [24]. In the present study, two anti-fungal *Lb*. *fermentum*-*S*.*cerevisiae* co-cultures were applied to 180-kg box fermentations to assess their influence on cocoa bean fermentation and drying processes and on final cocoa bean quality.

The inoculation with *Lb*. *fermentum*-*S*. *cerevisiae* co-cultures led to a dominance of *Lb*. *fermentum* M017 and 223, as revealed by colony morphology observations and qPCR analysis using an established method to detect *Lb*. *fermentum* and *Lb*. *plantarum* [25]. Higher concentrations of *Lb*. *fermentum* than of *Lb*. *plantarum* were found throughout inoculated fermentations, whereas during F-S, comparable concentrations of these two *Lactobacillus* species were detected (data not shown). During drying, colony observations on MRS plates and qPCR analyses showed no difference in LAB diversity between spontaneous and inoculated fermentations, suggesting that the inoculated *Lb*. *fermentum* strains did not dominate the surface of cocoa beans during drying. In terms of yeasts, the inoculated strain *S*. *cerevisiae* H290 did not seem to dominate the fermentation process, as suggested by colony morphology observations. Our findings correlate with the study of Miguel et al. (2017) [26] who detected a restricted species diversity of LAB but not of yeasts after inoculating a mixed culture of *Lb*. *fermentum* and *S*. *cerevisiae*, even though the cell concentration of the yeast inoculum, at 10^5^ CFU/ml, was ten times higher than that of the LAB inoculum, at 10^4^ CFU/ml.

The application of starter cultures to either speed up the fermentation process in the case of a co-culture consisting of *Saccharomyces cerevisiae*, *Lactobacillus plantarum*, and *Acetobacter aceti* [27] or to achieve a faster pH increase of the pulp upon usage of *S*. *cerevisiae* and *Torulaspora delbrueckii* [28] has been described. However, these effects were not observed in fermentations reported in this study. Inoculated cultures did not influence the microbial succession in terms of total aerobic counts, yeasts, and LAB, but led to slightly lower maximal AAB counts, indicating that the applied co-cultures suppressed outgrowth of AAB populations. The inoculated cultures may have led to lower oxygen levels in the pulp-bean mass negatively affecting strictly aerobic AAB [29], an assumption that is supported by the slower pulp degradation visually observed during inoculated fermentations. However, further research is needed to confirm the influence of the co-cultures on AAB counts.

The temperature measurements revealed the influence of a number of factors on temperature development in the centre of the fermentation mass. The inoculation with anti-fungal cultures appeared to slow down heat development, which is consistent with the slower temperature increase after the application of *T*. *delbrueckii* to the pulp-bean mass in the study of Visintin et al. (2017) [28]. The temperature in the centre of the fermentation mass was also affected by the position of the box in the fermentation building (Fig 1). Boxes that were placed close to the door exhibited lower temperatures, potentially due to increased air circulation and heat removal, than boxes in the back of the building. Closing the gaps between the wooden planks of the fermentation boxes for fermentation runs IIF and IIIF also led to higher temperatures and the elevated ambient temperatures in the first 4 days of IIIF further contributed to the fast temperature increase in IIIF. In general, the inhomogeneous distribution of the temperature in the fermentation mass, especially on fermentation days 2–3, highlights the importance of mixing the pulp-bean mass regularly to achieve a uniform fermentation of beans located in different parts inside of each fermentation box.

The temperature of the fermentation mass affected microbial successions in the fermenting pulp-bean mass. This was most obvious for yeasts, with counts dropping rapidly when temperature inside the fermentation mass rose above 45 °C, as described before by De Vuyst & Weckx (2016) [3]. In contrast to the fast decrease of yeasts during fermentations of IIIF, higher yeast counts were observed on the surface of drying beans in that run. A possible reason could be that thermotolerant yeast species developed at high temperatures at the end of fermentations during IIIF, e.g. *Pichia kudriavzevii* and *Trichosporon asahii*, which we had isolated previously from drying cocoa beans in Honduras [4]. The higher temperatures during IIIF caused a similar effect on LAB and total aerobic mesophilic germs with higher counts on dried beans after IIIF than after IF and IIF.

The courses of substrates and metabolites were comparable with only minor differences between inoculated and spontaneous fermentation processes, which is consistent with comparable substrate and metabolite levels detected in spontaneous and mixed-starter-culture fermentation by Crafack et al. (2013) [30]. More efficient utilization of pulp sugars and higher ethanol production in yeast starter culture inoculated fermentations reported by Ramos et al. (2014) [31] and Batista et al. (2015) [32], were only observed in terms of slightly higher ethanol concentrations in inoculated fermentations in this study. LAB counts on MRS were comparable for inoculated and spontaneous fermentations and lactic acid levels were not affected by the co-cultures. However, the dominance of *Lb*. *fermentum* strains M017 and 223 in inoculated fermentations led to increased pulp mannitol concentrations that can be ascribed to the ability of the heterofermentative *Lb*. *fermentum* strains M017 and 223 to produce mannitol as reported by Romanens et al. (2019) [19]. Differences were observed at strain level for the two *Lb*. *fermentum* strains, as higher acetic acid levels were found in pulp and beans fermented with *Lb*. *fermentum* strain M017 than in pulp and beans fermented with *Lb*. *fermentum* strain 223. This could be directly linked to the higher acetic acid production of strain M017 compared to 223 observed by Romanens et al. (2019) [19] when growing both strains in cocoa pulp simulation medium. No influence of the higher acetic acid levels on the cotyledon pH of inoculated beans was observed.

An increase in TPC measured during the fermentations in this study contrasts with a decrease in TPC from 161.1 mg/g on day 0 to 60.1 mg/g on day 6 during Nigerian cocoa bean fermentations [33], which may be explained by the use of different cocoa clones [34]. Losses of phenolic contents are caused by diffusion out of cotyledons and complexation reactions, while increased contents may be consistent with the formation of polymeric proanthocyanins [34], of which the latter might partially explain the increasing TPC content observed in the present study. Prolonged fermentations during IIIF seemed to affect the content of proanthocyanidins and (–)-epicatechin, as lower concentrations of these polyphenolic compounds were found after IIIF 8-day fermentations than after shorter fermentations of IF and IIF. This coincides with reduced proanthocyanidin contents after prolonged fermentations described by Oracz et al. (2015) [10]. Anthocyanins, which are responsible for the red colour in fresh bean cotyledons, usually disappear rapidly during the fermentation due to hydrolysis by glycosidases and are therefore markers for the degree of fermentation [23, 35]. Similarly, during the studied fermentations, anthocyanins disappeared after day 2, except for IF-B, where cyanidin-3-*O*-arabinoside was found on the last day of fermentation, which coincides with the low fermentation degree of these beans confirmed by the cut test. However, from the quercetin compounds that impart bitterness to cocoa [36], quercetin-3-*O*-arabinoside was not detected in samples from the final fermentation day, whereas quercetin-3-*O*-galactoside and quercetin-3-*O*-glucoside persisted throughout fermentation and drying. No influence of co-culture inoculation on alkaloid and polyphenolic contents could be observed.

In terms of dry bean quality, criteria for the cut test have been defined by Chocolats Halba (Wallisellen, Switzerland) with a minimum share of 75% for well-fermented beans and maximum shares of 3% for over-fermented and mouldy beans. The cut test confirmed that none of the fermentations yielded beans that fully achieved these requirements for quality A cocoa beans. Residual amounts of sucrose in dried beans after IF indicate incomplete hydrolysis of sucrose during fermentation by enzymatic and non-enzymatic reactions as described by Camu et al. (2008) [37] and correspond with the low fermentation degree revealed by the cut test. The lower amounts of well-fermented beans after inoculated fermentations is consistent with the slower heat development in these fermentations and indicates that co-cultures slowed down the fermentation processes. As pectinolytic yeasts have been described to speed up the fermentation process [30], the combination of the co-cultures with a starter culture yeast strain with pectinolytic activity could speed up the fermentation process and meliorate dried cocoa bean quality after inoculated fermentations. After IIIF-A and IIIF-B, the proportion of mouldy beans was above the threshold, which is consistent with the mould observed at the edges and sides of the fermentation boxes on days 7 and 8 during IIIF (data not shown). The development of mould might be linked to the low pulp acetic acid levels at the end of fermentation due to its evaporation, as acetic acid has been reported to prevent filamentous fungal growth due to its anti-microbial effect [38]. In addition to acetic acid, further anti-fungal metabolites may have been produced at low levels by LAB [39] or yeasts [40], but have not been analyzed.

Another crucial aspect of the quality of dry beans is their flavour profile. Low off-flavours, astringency, and bitterness and strong cocoa and fine flavours are generally acknowledged as desirable attributes by Chocolats Halba (Wallisellen, Switzerland). Despite differences in cotyledon pH, cotyledon metabolites levels, and cut test results of dried beans, the differences in sensory profiles perceived between the fermentations were small and the panel perceived no clear difference between inoculated and spontaneously fermented beans. Off-flavours were generally higher after the fermentations studied compared to the commercially fermented reference beans, which could be related to the higher acetic acid levels in the beans resulting from this study. Nevertheless, it was shown, that co-culture B led to less astringency and off-flavours and more cocoa flavours and acidity than co-culture A.

## Conclusion

This study, which assesses the influence of anti-fungal *Lb*. *fermentum*-*S*. *cerevisiae* co-cultures on the on-farm fermentation process, demonstrated great variability in the individual fermentation processes, highlighting the importance of applying culture strains at large scale and repeating experiments multiple times to obtain reliable data. The inoculated anti-fungal *Lb*. *fermentum*-*S*. *cerevisiae* co-cultures led to higher mannitol levels, due to the dominance of *Lb*. *fermentum* over *Lb*. *plantarum* in inoculated fermentations, and to slightly increased ethanol levels. Strain-specific differences were observed between the two *Lb*. *fermentum* strains, as beans inoculated with co-culture B including *Lb*. *fermentum* 223 exhibited lower acetic acid levels, higher proportions of well-fermented beans, less astringency, fewer off-flavours, and more cocoa flavours than beans inoculated with co-culture A including *Lb*. *fermentum* M017. For these reasons, co-culture B is suggested for future applications. Furthermore, compared to the spontaneous fermentation process, the anti-fungal co-cultures led to slower heat development and lower proportions of well-fermented beans. To overcome these negative aspects of the co-cultures and to speed up the fermentation process, combining the anti-fungal co-cultures with a pectinolytic yeast starter strain should be considered. Finally yet importantly, the anti-fungal activity of the *Lb*. *fermentum*-*S*. *cerevisiae* co-cultures against mycotoxin-producing filamentous fungal strains should be assessed by applying the co-cultures to a large number of on-farm fermentations, including fermentation processes under suboptimal conditions that favour mould growth.

## Supporting information

S1 Fig Workflow summarizing methods followed and main findings obtained during the present study. (TIF)

S1 Table Substrate concentrations in pulp and cotyledons during inoculated and spontaneous fermentation and drying processes of IF, IIF, IIF, and commercially fermented reference beans. Detection limits were at 0.2–0.8 mg/g for pulp samples and at 0.1–0.3 mg/g for cotyledon samples. Min. = minimal concentration during fermentation; Day = day on which the minimal concentration was detected: if the minimal value was recorded on more than one day, the first day with the same minimal value was listed; < = below detection limit; n.d. = not determined; n.a. = not applicable. ^a^One of two values below detection limit. ^b^Maximum instead of minimum is shown, as the value of day 0 was the minimal value or close to the minimal value.
(TIF)

S2 Table Metabolite concentrations in pulp and cotyledons during inoculated and spontaneous fermentation and drying processes of IF, IIF, IIIF, and commercially fermented reference beans. Detection limits were at 0.2–0.8 mg/g for pulp samples and at 0.1–0.3 mg/g for cotyledon samples. Max. = maximal concentration during fermentation; Day = day on which the maximal concentration was detected: if the maximal value was recorded on more than one day, the first day with the same maximal value was listed; < = below detection limit; n.d. = not determined; n.a. = not applicable. ^a^One of two values below detection limit.
(TIF)

S3 Table Sensory profiles of dried beans resulting from inoculated and spontaneous processes of IF, IIF, IIIF, and commercially fermented reference beans. ^a^To calculate the average ± standard deviation, values of seven panelists were included.
(TIF)
