# Supplementary figures and images for: Influence of two anti-fungal Lactobacillus fermentum-Saccharomyces cerevisiae co-cultures on cocoa bean fermentation and final bean quality
Source: PLoS One. 2020 Oct 1;15(10):e0239365. doi: 10.1371/journal.pone.0239365 (PMC7529286; doi:10.1371/journal.pone.0239365)

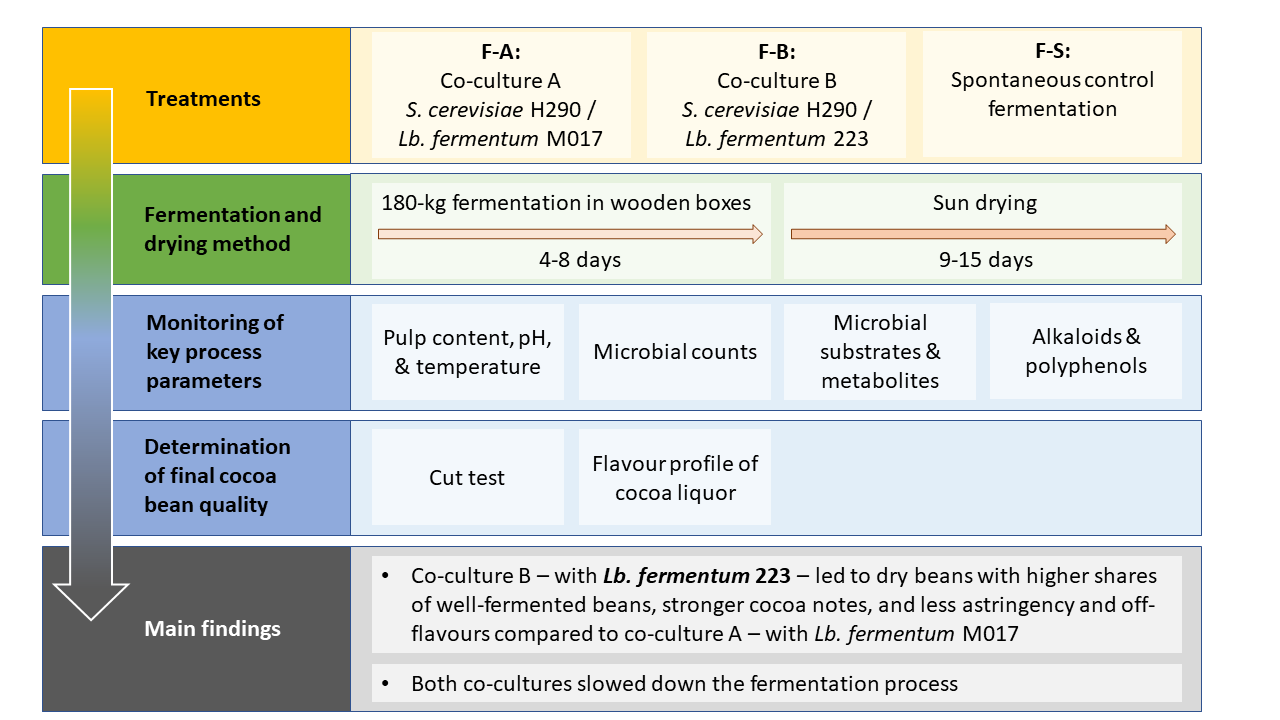

Supplement: S1 Fig — (TIF) [file pone.0239365.s001.tif]

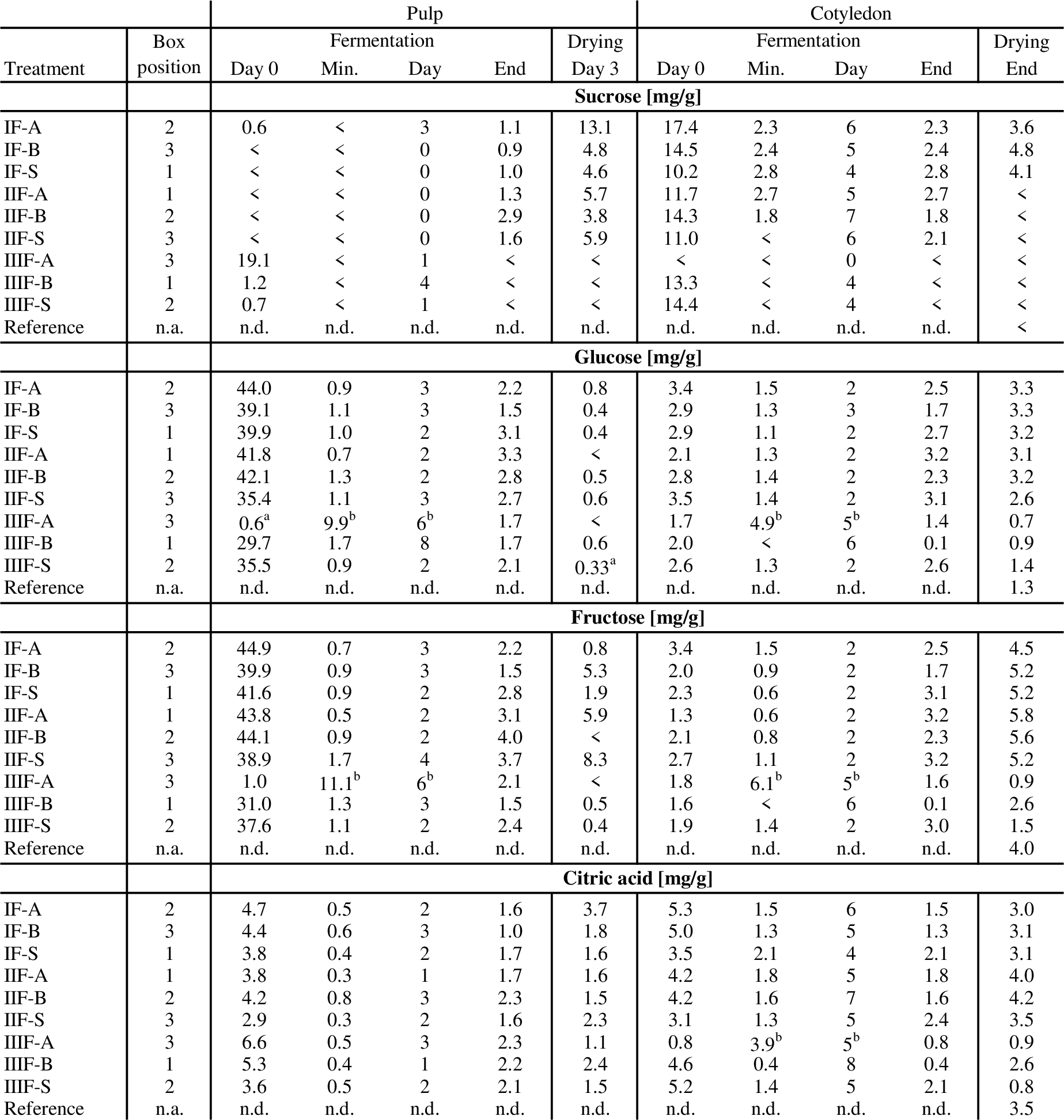

Supplement: S1 Table — Detection limits were at 0.2–0.8 mg/g for pulp samples and at 0.1–0.3 mg/g for cotyledon samples. Min. = minimal concentration during fermentation; Day = day on which the minimal concentration was detected: if the minimal value was recorded on more than one day, the first day with the same minimal value was listed; < = below detection limit; n.d. = not determined; n.a. = not applicable. aOne of two values below detection limit. bMaximum instead of minimum is shown, as the value of day 0 was the minimal value or close to the minimal value. (TIF) [file pone.0239365.s002.tif]

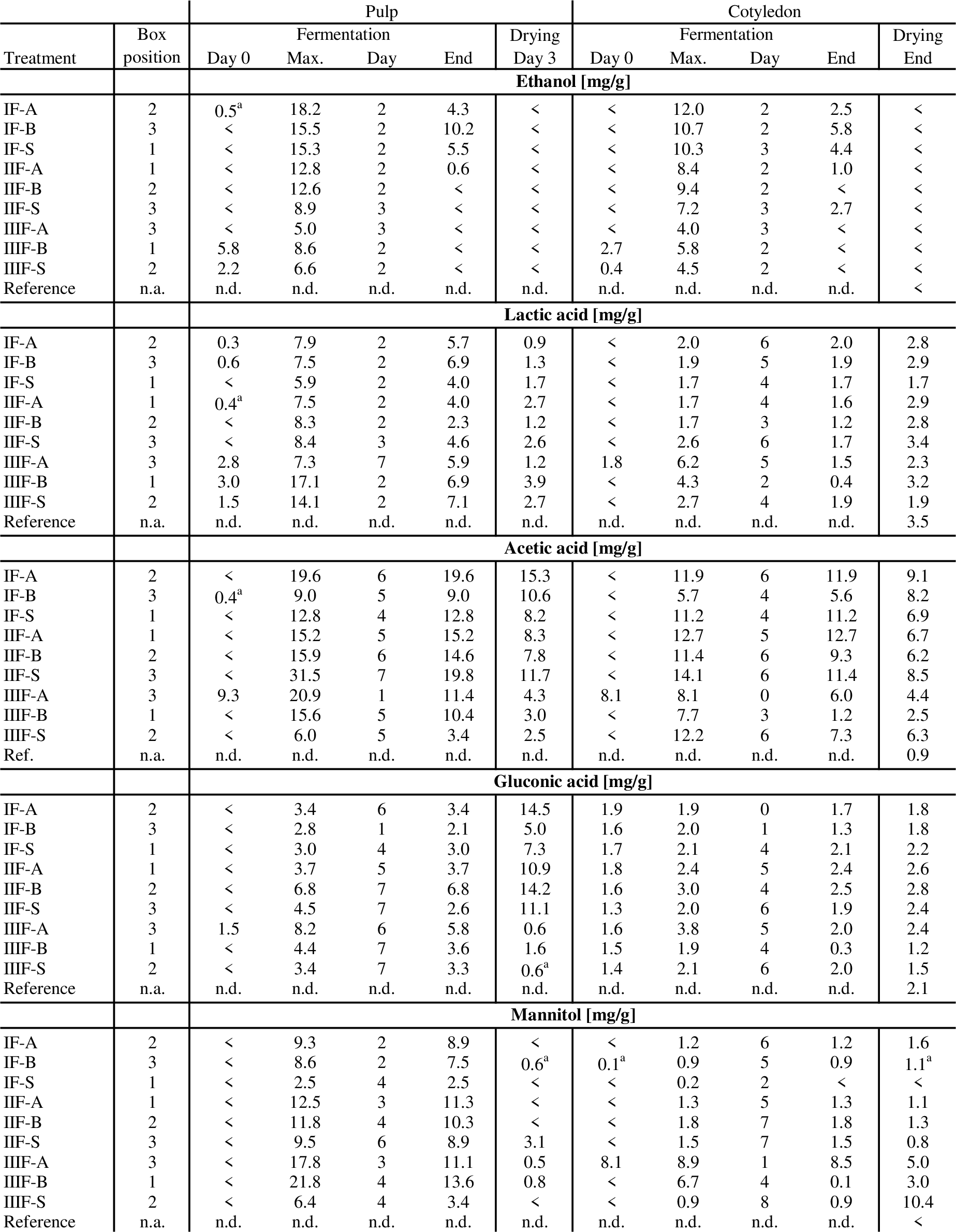

Supplement: S2 Table — Detection limits were at 0.2–0.8 mg/g for pulp samples and at 0.1–0.3 mg/g for cotyledon samples. Max. = maximal concentration during fermentation; Day = day on which the maximal concentration was detected: if the maximal value was recorded on more than one day, the first day with the same maximal value was listed; < = below detection limit; n.d. = not determined; n.a. = not applicable. aOne of two values below detection limit. (TIF) [file pone.0239365.s003.tif]

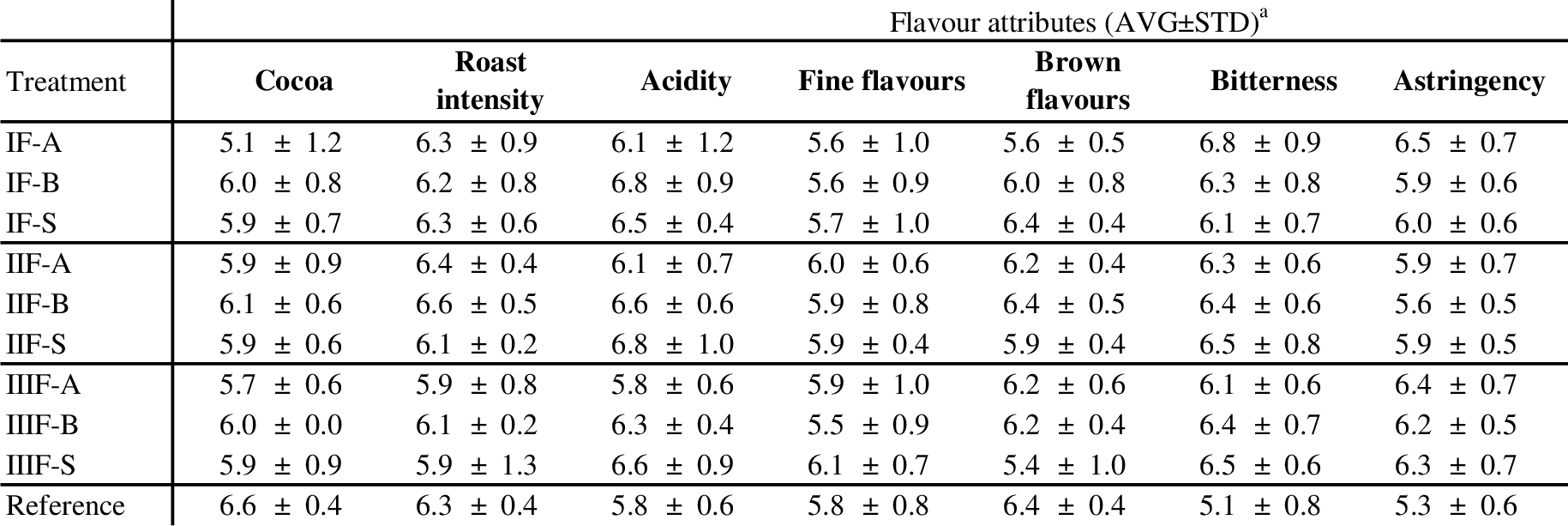

Supplement: S3 Table — aTo calculate the average ± standard deviation, values of seven panelists were included. (TIF) [file pone.0239365.s004.tif]
